# Conceptualization of Digital Platforms Within Cancer Communication: A Review of Barriers and Drivers of Online Tools to Empower Children and Adolescents with Cancer to Understand Their Disease

**DOI:** 10.3390/ejihpe15120242

**Published:** 2025-11-28

**Authors:** María Díaz-Cortés, Javier Morales-Mediano, Julio C. la Torre-Montero, Augusto Ferreira-Umpiérrez

**Affiliations:** 1 Department of Data Science (Quantitative), Universidad Loyola Andalucía, 41704 Sevilla, Spain; 2 Department of Marketing, Faculty of Economics and Business (ICADE), Comillas Pontifical University, 28350 Madrid, Spain; jmorales@comillas.edu; 3 San Juan de Dios School of Nursing and Physical Therapy, Comillas Pontifical University, 28350 Madrid, Spain; juliodelatorre@comillas.edu; 4 Fundación San Juan de Dios, 28016 Madrid, Spain; 5 Department of Well-Being and Health, Universidad Católica del Uruguay, Montevideo 11600, Uruguay; auferrei@ucu.edu.uy

**Keywords:** paediatric, oncology, digital communication, social media, mHealth, misinformation

## Abstract

The primary objective of this research is to identify the most influential factors in the digital platforms used by cancer patients and their environments for accessing oncopaediatric information. This is a PRISMA-guided systematic review that synthesises studies published between 2004 and January 2023 and does not report new primary data. We explore the drivers and barriers of web-based platforms, health apps and social media. We conducted a literature review guided by the PICOS strategy: (P) children and adolescents; (I) factors affecting the use of health apps and social media; (C) without a specific comparison; (O) measuring impact, understanding and success factors; (S) using a conceptual approach. Our study reveals a dual dynamic in paediatric oncology science communication, in which drivers (information, collaborative efforts, comprehensive education) and barriers (age-appropriate content, misinformation) shape the complex communication landscape. The reality is that a healthcare application is needed that focuses on extensive education and the paediatric patient’s involvement in understanding and improving their well-being. It requires adapting communication strategies. Additionally, we explore the theory of online health communication and identify several promising avenues for research.

## 1. Introduction

Chronic diseases, such as cancer, are life-limiting and significantly affect the patients and their families (D. Hansen et al., 2016) by causing deep and lasting changes in daily lives (Heilferty, 2018). In children and adolescents, this impact becomes even more complex (Levit et al., 2014).

Despite advances in cancer treatment, communication in paediatric oncology remains an underexplored yet crucial dimension of care. Both patients (Civan et al., 2009) and healthcare professionals (Perales et al., 2016) have expressed the need for reliable, accessible tools that support information exchange (Schiffman et al., 2008). Digital communication has transformed the traditional flow of information, and social networks now play a pivotal role in enabling interaction and emotional support. As a result, healthcare professionals are increasingly positioned to guide and supervise these digital exchanges, ensuring that information is accurate, age-appropriate, and beneficial for young patients and their caregivers (Stiles & Mynard, 2021).

This study presents a systematic review of the literature conducted between 2004 and 2023, aiming to identify and synthesise the main factors that facilitate or hinder digital communication in paediatric oncology. Rather than offering an extensive review of previous work in this section, the analysis of the literature and thematic findings are developed in later sections of the paper.

## 2. Background and Literature Review

The following subsections constitute the literature review that supports this study.

### 2.1. Scientific Information for Young People with Cancer

Young people affected by cancer must acquire knowledge and skills in managing their illness if they are to have a positive impact on the course of their disease (Civan et al., 2009). Barlow et al. (2007) state that individuals who understand their illness and its treatment can better manage both their physical and emotional health. This is because they are better prepared to cope with emotional grief and the difficult decisions they must make following a cancer diagnosis (Schroeder et al., 2021). However, becoming informed can also have adverse effects. Civan et al. (2009) claim in their study that, for some patients, the consumption of information generates stress and anxiety. This aspect requires further study, as it may be influenced by the type of aggressiveness and stage of the disease (Landier, 2011). Let us look at this in more detail.

Firstly, the stage of the disease affects (1) the frequency with which patients’ families consult information sources, (2) the type of information they seek, and (3) the involvement of educational programmes in the hospital stage of the disease (Landier, 2011).

Secondly, it is essential to highlight that all the aspects mentioned above have a common problem: the need for more adaptation of language and format for children and adolescents (Gilljam et al., 2020). The main reason for this problem is that the scientific community and health professionals use technical terms to communicate with them (Domínguez & Sapiña, 2014). To address this, federal funding agencies, such as the N.I.H. and the Patient-Centred Outcomes Research Institute, began calling for greater involvement of young people or their guardians in finding solutions for them (Forsythe et al., 2016), but the problem persists.

### 2.2. Health and Digitisation

Digital health is an alternative that promotes the personalisation of care and empowerment through knowledge (Delemere et al., 2022). This concept, defined as healthcare that links people, processes, and technology (Barr et al., 2014) through technological mechanisms such as sensors, mobile devices, and health (Iglehart, 2014), promotes participation in decision-making about illness. Its aim is not to supplant the patient-health professional relationship, nor to reduce the latter to unreliable sources of information (Hesse et al., 2010; C. Lee, 2008; Murray et al., 2003), but to promote online interactions (McMullan, 2006). Through this, individuals can access, transmit, or receive health information, guidance, or support (Patrick, 1999).

Another positive aspect of the digital health phenomenon is how it helps address the medical, personal, or social uncertainty caused by a cancer diagnosis. In the health context, uncertainty is a person’s perception that they cannot make sense of information or circumstances related to the disease (Ussher et al., 2006). Technologies can manage this uncertainty through interactions between individuals (Patrick, 1999) and by unifying a patient’s knowledge and health history, which can then be updated and consulted by multiple professionals (Delemere et al., 2022).

### 2.3. Digital Health and Children with Cancer

The digital revolution has played a significant role in the advancement of childhood and adolescent cancer. When used effectively, digital health is a valuable communication tool that can be tailored to the needs of children and adolescents, enabling them to describe their experiences, facilitate decision-making, and engage in developing their disease management (Wyatt et al., 2015).

### 2.4. A New Direction for Digital Health

This new era in the healthcare system (Ruland et al., 2008) enhances healthcare professionals’ knowledge, skills, and training in diagnosing, treating, and caring for patients (Martiniuk et al., 2021). However, the barriers to healthcare posed by the new era must be analysed (Grace et al., 2019).

At the time of this study, no research explicitly focused on the drivers and barriers of digital platforms on which paediatric oncology-related health content is communicated by incorporating online communication theory. For this reason, the present study aims to (1) explore the most influential characteristics of digital platforms and their interconnection with health communication and (2) provide scholars and practitioners with a suitable reference to frame the main scientific questions related to digital health communication to identify new avenues of research. Thus, collaborating with the third, fourth, and tenth goals outlined by the World Health Organisation (WHO): (1) ensuring healthy lives and promoting well-being for all; (2) providing inclusive and equitable quality education; and (3) reducing inequalities (Gámez, 2022; García, 2023).

### 2.5. Theoretical Background

With the emergence of digital health platforms, paediatric oncology science communication is undergoing a significant transformation (E. Lee et al., 2022), offering new ways to engage and empower patients. Engagement is reflected in the increasing trend among patients to use the Internet as a primary source of health information (Tan & Goonawardene, 2017). Indeed, several studies have examined interactions among patients exchanging health information online (Chung, 2013; Luo et al., 2022; Wald et al., 2007).

To better understand this concept, we utilise the theory of online health communication. It considers the interaction between scientific communication, digital platforms and patients’ needs (Heilferty, 2009). It explains how technology and the Internet have transformed the way people access, share, and use health information, how they influence health-related decisions and behaviours, and how health professionals can utilise these communication channels effectively.

Heilferty (2009) exemplifies online communication theory by drawing on the structure and dynamics of disease blogs, which have become invaluable tools for scientific communication (Heilferty, 2009). In this study, we extrapolate this to digital platforms, such as Facebook groups among many others, as they are now great repositories of first-hand accounts in which medical knowledge and emotional narratives can also appear (Linn et al., 2023) and create a common thread between scientific discourse and the lived experiences of paediatric cancer patients (Duimel et al., 2022; Yli-Uotila et al., 2013; Zufferey et al., 2010).

This study primarily utilises online communication theory to facilitate the navigation and dissemination of health information online, thereby bridging the gap between clinical consultations and digital resources (Heilferty, 2009). Its application plays a role in patient care for those who are ill, as it enables the development of strategies to effectively disseminate scientific content by empowering and reassuring patients and their families through comprehensive and understandable information (Heilferty, 2018). Cancer patients require an interaction-based model centred on comprehensive knowledge (Bluebond-Langner, 2000) that incorporates scientific content and resonates with their unique experiences and emotions, thereby facilitating their understanding (Heilferty, 2009).

In the dynamic landscape of paediatric oncology, incorporating online communication theory is not just an option but a necessity. With these considerations in mind, we set out to answer the following research questions (R.Q.):

*RQ1:* What key drivers or facilitating factors enhance online scientific communication in paediatric oncology, and are they reported across the reviewed studies?

*RQ2:* What barriers or challenges are identified in the literature?

*RQ3:* How do these drivers and barriers differ across the leading digital platforms used in paediatric oncology communication?

## 3. Materials and Methods

This section presents the PRISMA-guided methodology employed in the review, encompassing the search strategy, eligibility criteria (PICOS), screening, data extraction, and thematic synthesis. The subsequent qualitative analysis examines the bibliographic data collected using the PICOS framework (for defining the search strategy) and PRISMA guidelines (for article selection). Specifically, we detail below the analytical model, coding steps, coder roles, reliability checks, and iteration cycles that structured the inductive thematic synthesis (Thomas & Harden, 2008; Braun & Clarke, 2006). The objectives of this methodological approach are to (i) identify and synthesise the drivers that facilitate digital scientific communication in paediatric oncology, (ii) identify and synthesise the barriers that hinder it, and (iii) compare how these drivers and barriers manifest across platform types, including websites/portals, mobile applications, and social media. This systematic review has been registered in PROSPERO under the registration number CRD42025117771.

### 3.1. PICOS Method

The PICOS method enables the definition of specific information (Landa-Ramírez & De Jesús Arredondo-Pantaleón, 2014) by allowing the researcher to determine the criteria for including and excluding studies, terms, and databases to answer their research questions. This approach enables the researcher to structure the work effectively, and the literature search focuses on the most suitable interventions. Its strategy has four or five questions (Landa-Ramírez & De Jesús Arredondo-Pantaleón, 2014).

*Population:* Cancer patients aged 5–17 years, guardians and healthcare staff.

*Intervention:* Identify the factors that affect the use of health apps and social networks for educating oncology patients and adapt the tools and resources accordingly.

*Comparison:* Not applicable

*Results:* Impact, scientific understanding, satisfaction, education, usage, success factors, digital platforms, language, linguistic formats, communication, generations, and colours.

*Study design:* Systematic search of existing information and conceptualisation of the technological world within paediatric oncology.

Having clarified the PICOS questions and the research objective described in the introduction, the inclusion and exclusion criteria were established (see Table 1). In this way, we established our final search strategy.

### 3.2. PRISMA Method

After establishing the initial inclusion and exclusion criteria, keywords were selected to determine our search strategy. For this, we used PRISMA (Page et al., 2021) (Preferred Reporting Items for Systematic Review and Meta-Analysis), a method whose process ensures rigour, transparency and reproducibility by improving research quality, minimising bias and facilitating informed evidence-based decision-making (Haddaway et al., 2022).

We selected Web of Science (WoS) as our information source. WoS is the most appropriate database for the specific research objectives, as it provides extensive citations and metrics while maintaining a consistently high reputation and quality as a bibliographic repository (Zhu & Liu, 2020).

*Search strategy*

Based on our inclusion and exclusion criteria, we established and introduced our WoS search strategy on 17 January of 2023 as follows:

(“social media” OR “Instagram” OR “Twitter” OR “Facebook” OR “TikTok” OR “popular online platforms” OR “mass media” OR “mHealth” OR “microblogging network” OR “social networks” OR “networks” OR “digital” OR “computer-mediated”)

AND

(“communication” OR “education” OR “understanding” OR “dissemination” OR “information exchange” OR “information seeking”)

AND

(“oncology” OR “cancer” AND “teenager” OR “parent” OR “AYAs” OR “adolescent” OR “caregiver” OR “paediatric” OR “paediatric”).

*Selection criteria*

We chose thematic areas related to medicine, science, communication, education, technology, and humanity because the research addressed all of them.

On the other hand, we checked which journals were clearly in J.C.R.J.C.R. to ensure their quality. As a result, we obtained the following:

- International Journal of Environmental Research and Public Health.
- Medical research journal on the Internet.
- B.M.C.B.M.C. Public Health.
- Plos One.
- Cochrane Database of Systematic Reviews.
- Journal of Health Communication.
- Frontiers of public health.
- B.M.J.B.M.J. Open.
- Health communication.
- Social Sciences and Medicine.

The publication phase should include articles with complete and proven results, so they must be in the final phase. In addition, we wanted up-to-date information, so we limited our search to the years between 2004 and 2023, and from there, we used the most relevant cross-references.

With all these filters, we obtained 578 articles. After peer review and blind analysis using the Rayyan platform (an intelligent, timesaving, collaborative research platform for literature reviews and systematic reviews), two reviewers selected a final set of 75 articles.

**Figure 1:**
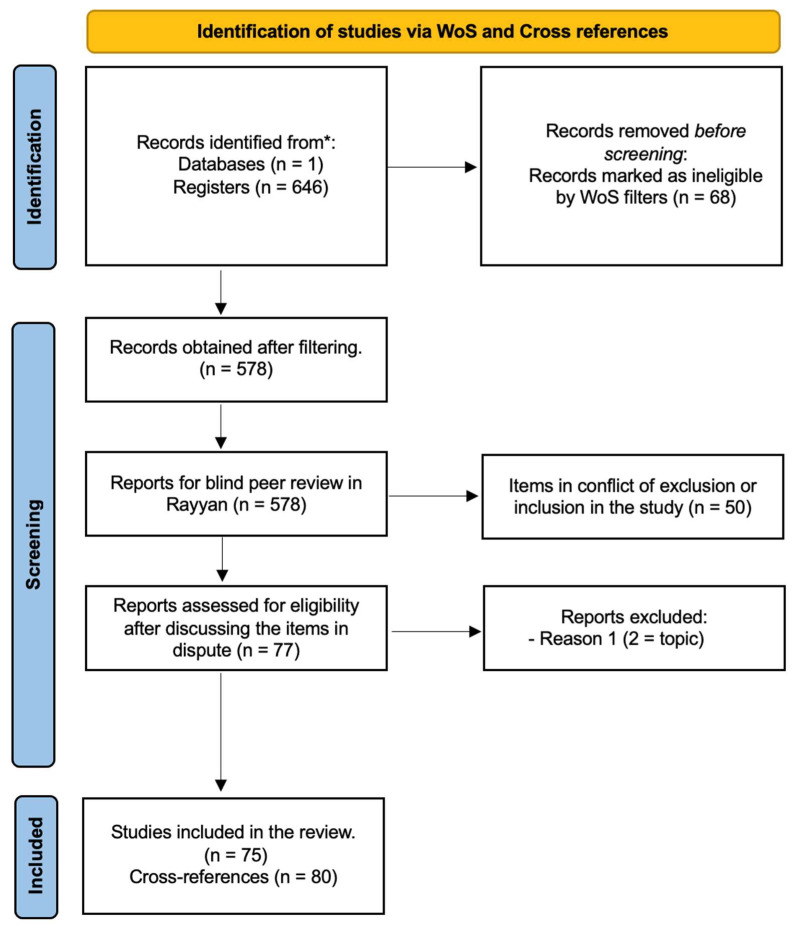
PRISMA flow (own elaboration based on Page et al., 2021).

During the selection process, the PRISMA flow diagram (Figure 1) summarises the progressive screening of studies. In the first screening, 68 articles were excluded because their focus was not cancer-related but instead addressed other health topics, such as COVID-19 and non-oncological diseases. In the second screening, 50 articles were excluded because they did not meet the thematic requirements outlined in the inclusion criteria table (Table 1), particularly regarding the Target, Intervention, Comparator, and Outcome dimensions. In the third and most extensive screening, 451 records were excluded as they referred to adult oncology (e.g., tobacco-related, ovarian, or prostate cancers) rather than paediatric oncology. As a result, 75 studies were retained for qualitative synthesis, and additional papers were incorporated through cross-referencing.

Each article was independently reviewed by two researchers using these predefined inclusion and exclusion criteria. Discrepancies between reviewers were discussed in consensus meetings until agreement was reached, and when necessary, a third researcher mediated the decision. This multi-step, consensus-based process ensured consistency and minimised potential selection bias throughout the screening phase. To ensure reliability and transparency, the screening and synthesis process followed the PRISMA 2020 guidelines. Each study was independently reviewed by two researchers, with discrepancies resolved through discussion and, when necessary, arbitration by a third reviewer. This multi-step consensus approach minimised potential selection bias and enhanced the reproducibility of the review.

### 3.3. Inductive Thematic Synthesis and Triangular Conceptual Framework

The inductive thematic synthesis was applied from an interpretative-constructivist perspective, aiming to generate transversal meanings across heterogeneous studies and construct theoretical explanations of digital communication processes in paediatric oncology. This perspective assumes that knowledge is co-constructed from the contextual interpretation of the researcher and empirical data (Braun & Clarke, 2006; Thomas & Harden, 2008). The objective was to transcend the mere description of individual results and identify relational patterns, shared meanings, and underlying mechanisms that shape digital communication among paediatric patients, families, and health professionals.

The process integrated qualitative and quantitative evidence from studies included in Rayyan, in coherence with the convergent synthesis paradigm for mixed-methods research (Sandelowski et al., 2006). To ensure methodological rigour, the analysis followed internationally recognised guidelines, particularly Braun and Clarke (2006) and Thomas and Harden (2008), complemented by transparency and auditability criteria recommended by Nowell et al. (2017).

The synthesis was developed through four iterative stages:

- Familiarisation and line-by-line inductive coding, performed independently by two reviewers in Rayyan using a blinded system to reduce bias and strengthen analytical independence (Nowell et al., 2017). The outcome of this inductive coding process is summarised in the final codebook (see Table 2).

- Comparison and clustering of emergent codes during consensus meetings, with discrepancies resolved by a senior third reviewer to reinforce analytical reliability. The main findings from the analysed studies were further synthesised in a comprehensive matrix, allowing for a visual inspection of the thematic and methodological distribution across the sample (see Table 3).

- Cycles of iterative refinement with recoding, redefinition of categories, and constant comparison between codes and studies to ensure theoretical saturation and internal consistency (Sandelowski et al., 2006).
- Systematic documentation of analytic memos, coding logs, and grouping matrices, ensuring process traceability and transparency in interpretative reasoning (Lincoln & Guba, 1985).

**Figure 2:**
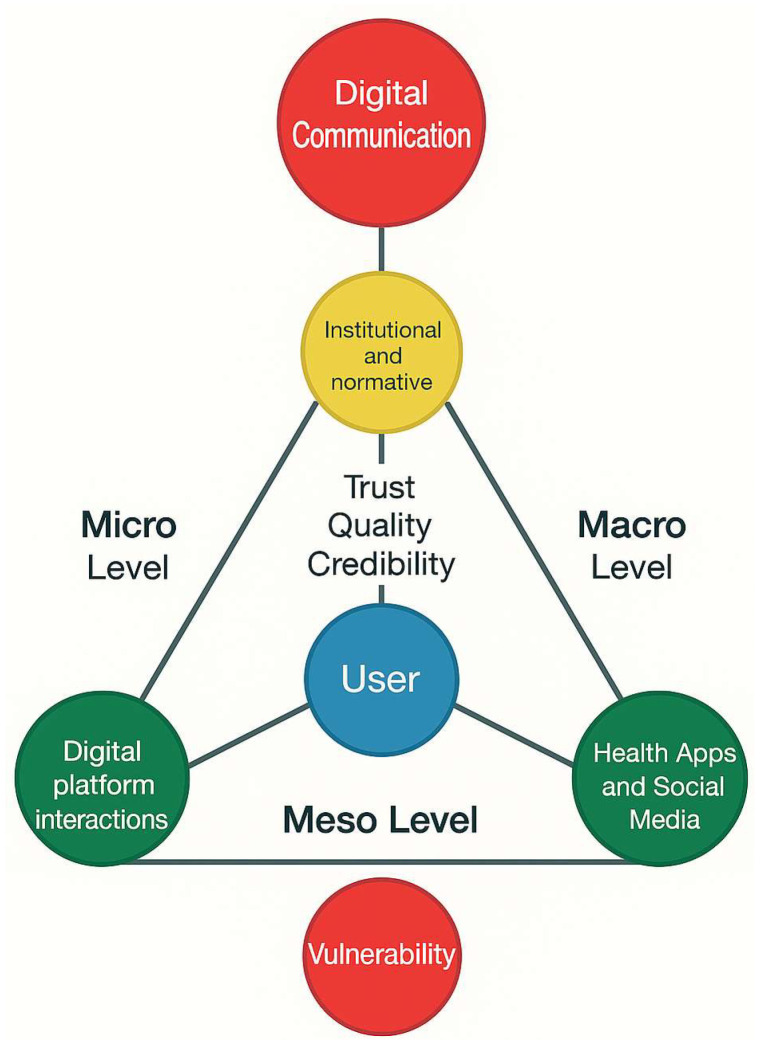
Triangular Conceptual Framework of Digital Communication Across Micro, Meso, and Macro Levels (Own elaboration, 2025).

The procedure was entirely inductive, with no prior analytical framework, and no qualitative software was used beyond the Rayyan platform. All coding, thematic construction, and interpretation decisions were documented in audit logs and maintained as Table 2 and Table 3 and Supplementary Material (PRISMA-ScR). Inter-coder reliability was estimated to be greater than 90%, with divergences resolved through consensus or expert arbitration. This approach enabled the development of a triangular conceptual framework (see Figure 2) articulating the micro (digital platform interactions), meso (family and clinical team dynamics), and macro (institutional and normative structures) levels of the studied phenomenon, situating digital communication within a broader relational ecology.

To clarify this analytical framework, it is first necessary to explain how the two categories—drivers and barriers—were conceptualised and operationalised in the study. We define a driver as an element that enhances communication effectiveness and promotes awareness and collaboration among healthcare professionals, young patients, and their families (Khair et al., 2011). As the study focuses on online communication, technological advances, educational initiatives, and empathetic communication strategies tailored to the specific needs of paediatric cancer patients (L. Cheng et al., 2021; Milne-Ives et al., 2020a) are also considered potential drivers.

Barriers, on the other hand, are those that impede the flow of scientific information and hinder informed decision-making by patients and their caregivers. These barriers may include inadequate educational resources or systemic deficiencies in healthcare communication protocols (Sedrak et al., 2018). Identifying and understanding these barriers is crucial to fostering children’s and adolescents’ engagement in their treatment through a more comprehensive understanding of scientific content (Barlow et al., 2007).

Once these conceptual definitions were established, all 75 articles retained through PRISMA screening were analysed using the inductive thematic synthesis described above to empirically identify how these drivers and barriers manifest in the literature. Following independent full-text readings by two reviewers, recurrent concepts and keywords were extracted and discussed in consensus meetings. This process yielded two overarching analytical dimensions—drivers and barriers—within which seven recurrent themes were identified. The drivers most frequently reported across the reviewed studies included social media, educational or training initiatives, and collaborative or empathic communication. The most commonly cited barriers included misinformation, lack of trust or credibility, excessive technical language, and insufficient age-appropriate adaptation. These categories were not predefined but emerged inductively from the literature. Table 3 provides a synthesis matrix summarising the thematic distribution of each theme across the included studies.

Having explained these two concepts, it is important to explore them further and analyse their correlation with the empowerment of young people facing the challenges of paediatric oncology.

## 4. Results

Our study conceptualises the main drivers and barriers of online scientific communication in paediatric oncology, establishing how these two analytical categories interconnect to shape young people’s understanding and emotional engagement with their treatment.

Building on the methodological process detailed in Section 3.3, the following results present the analytical findings of the inductive thematic synthesis, highlighting how drivers and barriers materialise across the literature. The conceptual definitions and coding framework are summarised in Table 2 and Table 3 (Methods section), while the interpretative insights are described below.

Two overarching analytical dimensions—drivers and barriers—emerged from the synthesis, encompassing seven recurrent themes that capture the dynamics of digital communication among patients, families, and healthcare professionals.

### 4.1. Drivers and Barriers to In-Depth Science Communication

Social media emerged as the most recurrent and influential driver of scientific communication across the reviewed studies, according to the thematic frequency analysis. This finding aligns with the observation that social networks function as dynamic spaces for cancer-related discussions, creating repositories of information and diverse data exchanges. Different types of social media platforms expand the availability of health information sources (Yıldırım et al., 2018) and generate large volumes of content that empower patients through access to shared knowledge (Grace et al., 2019). Table 4 provides a detailed overview of the topics and coding categories identified in the analysis of medical information exchanged through social networks.

Another feature of social networks is that they reduce the complexity of health communication and improve its effectiveness. The complexity of health communication stems from the fact that health professionals’ main work is primarily practical rather than communicative. However, the natural property of social media is to facilitate in-depth discussions (Marcus, 2014). This property provides patients and their entourage with alternatives to learn about different treatment options, reduces the frustration and burnout of healthcare professionals, and generally improves patient satisfaction (Rupert et al., 2016).

Finally, it must be acknowledged that social media have the potential to address the lack of information tailored to children and adolescents (Ruan et al., 2020). However, it is not just a matter of providing a large amount of child-friendly, accessible information, but also of considering critical factors such as quality, credibility, and the language used, among others. Although standards have already been outlined to assess some aspects, such as the quality of online medical data (Martiniuk et al., 2021), significant discrepancies remain in these areas. Therefore, the following section addresses the challenges the scientific community will face.

### 4.2. Challenges for Scientific Research

This section will analyse misinformation, mistrust, lack of credibility, and the use of technical language as obstacles. These specific obstacles were selected because they consistently emerged as dominant barriers across the thematic synthesis, with misinformation, mistrust and credibility issues, and overly technical language. Their recurrence across multiple contexts justified their inclusion as core analytical categories.

We start with inaccurate information. It is a detrimental obstacle to the development of decision-making processes for patients and their environments, which we find particularly pronounced on social media (Gage-Bouchard et al., 2017, 2019). It is due to the high prevalence of misinformed people discussing health issues on these platforms, which has serious consequences that can lead to poor decision-making in treatment choices that subsequently generate emotional distress (Loeb et al., 2019).

We continue to build trust and credibility, two concepts that are distinct yet closely related. They are fundamental elements for effective communication, but on many occasions, they must be present in social networks, significantly limiting good communication. There is an urgent need to address this barrier, as patients say they need more confidence in the information they find, as it generates anxiety and uncertainty (Delemere et al., 2022).

The last barrier we want to address in this section is the use of technical language. Patients and their environment are generally unfamiliar with medical terminology and require assistance in understanding it. Their lack of understanding can lead to misunderstandings, difficulties in patient-health professional communication and relationships, and slow decision-making (Farnan et al., 2013).

We conclude that science communication in health is a complex interplay between drivers and barriers. There is still much room for improvement in information contamination, lack of age-appropriate content, misinformation, trust deficits, and language barriers.

### 4.3. Types of Social Networks Where Paediatric Oncology Is Discussed

In health communication through online media, the drivers and barriers materialise. Therefore, it is essential to be aware of them and understand why they have emerged. Below, we examine leading social networks used for paediatric oncology information to identify their characteristics and their effects on patients and their environments.

Social networks are virtual spaces that have evolved into robust networks providing news, psychosocial support, tangible resources, and financial assistance (Brown, 2020). This transformation has driven a shift in healthcare, moving it into an increasingly digital realm (Milne-Ives et al., 2020b; Lam et al., 2013) and even healthcare itself (M. Cheng et al., 2021).

Social networks, therefore, represent a technological advancement that enables health promotion, prevention, treatment, and maintenance (Milne-Ives et al., 2020b), effectively overcoming geographical and temporal limitations (Wenzel, 2018).

In recent decades, health communication researchers have shown interest in three categories of digital platforms: web-based platforms, health apps and social platforms. Each platform assumes a unique position about end users and shapes the landscape of science communication, as illustrated below:

### 4.4. Health Websites for Support and Training

These pages are dedicated to uploading valuable resources on paediatric oncology and serve as a repository of scientific information (Love & Donovan, 2013; Yli-Uotila et al., 2013). Among the content in these resources is structured educational material, which is essential for improving comprehension.

This type of website often employs technical language, but its content is generally credible and reliable, as it originates from official institutions (Domínguez & Sapiña, 2014). There are several types of websites: (1) focused on collaborative projects or (2) websites of governmental organisations and societies such as the American Cancer Society (Grace et al., 2019). See Table 5 to understand the most influential aspects of scientific communication on these platforms.

### 4.5. Mobile Health Applications (mHealth) in Paediatric Oncology

mHealth is a subset of digital health that delivers health-related information and services through mobile communications and wearable devices (Källander et al., 2013). These applications are closer to end-users than web platforms, providing real-time information and resources (Doorenbos et al., 2020). However, they still maintain a significant distance from paediatric and adolescent audiences. Most health apps supposedly designed for children and adolescents with cancer often lack a direct approach to this audience and seem more tailored to parents (Lam et al., 2013).

These software programmes designed for mobile devices play a crucial role in healthcare (Van Velthoven et al., 2018) as they are tools that serve as educational resources for patients by allowing them to monitor their health data and manage their symptoms, modify their behavioural habits, and improve their preparedness for disease (Cunningham et al., 2021) and decision-making (Mueller et al., 2018).

The advantage of such apps is that they empower patients by enhancing their skills and confidence in managing their medical condition (Wittmann-Price et al., 2012). This active methodology enables patients to gain a deeper understanding of themselves and, consequently, participate more actively in their care (Berkowitz et al., 2017; Jongerius et al., 2019). It does this through:

- Improved management through support in physical assessment, follow-up procedures, medication dosage and related healthcare issues (Doorenbos et al., 2020). This multifaceted approach ensures that users are well-equipped with the knowledge and skills needed to navigate the complexities of healthcare (Becker et al., 2014; Birkhoff & Smeltzer, 2017).
  Its real-time monitoring tools enable the monitoring and management of health symptoms (Doorenbos et al., 2020; Mueller et al., 2018). Moreover, the medication assistance function sends reminders to promote medication adherence and ensure timely administration of medication (Berkowitz et al., 2017; Jongerius et al., 2019).
- They positively influence patient care (Jongerius et al., 2019), particularly in cancer treatment (Berkowitz et al., 2017), by facilitating continuous and active patient engagement throughout the treatment process. This involvement fosters a collaborative healthcare environment (Ostrovsky & Chen, 2020), resulting in improved patient outcomes and a more patient-centred approach to care (Berkowitz et al., 2017).
- Their innovative features (see Table 6).

In conclusion, these applications have training and support functions that encourage patient participation and promote holistic health management. Both aspects provide comprehensive patient education, making these apps powerful allies in health promotion, disease management, and the pursuit of a better quality of life.

### 4.6. Social Platforms

It is a highly interactive, multidirectional tool that provides informational and emotional support and has transformed how people consume information (Myrick et al., 2015). Its success lies in the tone used to disseminate content, the immediacy of the response, the relevance of the information and the creation of personalised information communities (Domínguez & Sapiña, 2016).

As it enables high user interaction and has a substantial impact on society (M. Cheng et al., 2021), examining its drivers and barriers to usability among children and adolescents with cancer is necessary.

In healthcare, social platforms must address misinformation, ensure information accessibility, and create high-quality content for a vulnerable audience (Gage-Bouchard et al., 2019). It is now possible for patients and their caregivers to self-educate and communicate with people who share similar clinical experiences. It is known as the democratisation of health information, and while it removes barriers to access, it shifts consultations with clinicians (Metzger et al., 2010). Thus, the influence of social networks on the doctor-patient relationship (Metzger et al., 2010) has undergone significant changes. Due to this change in relationships, social platforms must be constantly updated and ensure they do not lead to a deterioration in medical care, but rather to improvements, such as collaboration with accredited healthcare professionals (Kivits, 2009; Powell et al., 2003). Refer to Table 7 for details on the platforms and their characteristics.

In summary, the role and impact of these social platforms in transmitting scientific knowledge to children and adolescents fighting cancer are paramount in paediatric communication. Understanding and exploring the drivers and barriers should encourage the development of these platforms and ultimately have a positive impact on patients and caregivers. However, it remains imperative to understand how these digital platforms exhibit distinct dynamics in user interactions (Gage-Bouchard et al., 2017).

## 5. Discussion

Research recognises the transformative impact of digital platforms in science communication for children and adolescents facing cancer. Having analysed previous studies on patient interactions in online health information exchange, we conceptualise in this article the interplay between science communication, digital platforms, and the unique needs of paediatric oncology patients by addressing barriers and leveraging communication drivers.

Because this study adopts a PRISMA-guided systematic review design, the following discussion integrates evidence drawn from previously published research rather than presenting new empirical data. Accordingly, the analysis should be understood as a synthesis of recurrent patterns and conceptual trends identified across the reviewed studies, offering a comprehensive perspective on the drivers and barriers shaping digital communication in paediatric oncology.

Research by E. Lee et al. (2022) demonstrates the relevance and applicability of online communication theory in informing strategies that empower and reassure cancer patients and their families by providing accessible scientific information. Furthermore, the study highlights the role of online communication theory in tailoring content to the specific needs and motivations of paediatric oncology patients, ultimately fostering a deeper and more meaningful understanding of scientific information. This research enriches the theoretical foundations of online health communication and illustrates its practical utility in optimising science communication for the unique challenges of paediatric oncology patients.

Building on these theoretical insights, our review specifically contributes to identifying how these dynamics operate within paediatric oncology, a domain where evidence remains limited and fragmented. To operationalise these theoretical perspectives, we structured our analysis around three research questions that examine the key drivers, barriers, and platform-specific characteristics shaping online scientific communication in paediatric oncology.

Our study contributes to understanding the influential aspects that shape the success of online scientific communication in paediatric oncology. In response to the research questions, we can highlight that:

In response to RQ1, the study identifies key drivers of effective online communication. It recognises the role of social media in empowering patients (Warner et al., 2021) through information, as they serve as platforms for cancer-related discussions and generate a wealth of information. Additionally, research highlights the importance of simplifying complex knowledge to enhance communication effectiveness. By analysing the interplay of these factors, the study provides insights for future research, enabling the development of personalised communication strategies that engage and empower young patients facing the challenges of paediatric oncology.

Turning now to answer RQ2, the study identifies barriers to online scientific communication in paediatric oncology. In particular, the prevalence of misinformation on digital platforms stands out as a substantial risk to the decision-making processes of children and adolescents facing cancer. Trust and credibility deficits are also flagged as critical barriers, inducing anxiety and uncertainty among paediatric patients. The study further identifies technical language in health information as a significant barrier, emphasising the need to create accessible and age-appropriate communication, as proposed by Cowfer et al. (2023). With this knowledge, future studies can address these challenges, improving the effectiveness of online scientific communication and patient engagement in paediatric oncology.

For the last R.Q. 3 in the study, we uniquely examined how drivers and challenges manifest across the various digital platforms used in online health communications related to paediatric oncology. To do so, we analysed websites, health apps, and social networks and delineated the distinctive characteristics of each platform. As a result, we identified the need for an educational, multilingual, and interactive approach to enhance understanding and promote a global community. This approach would remove age-specific barriers and mitigate the risk of misinformation.

To contextualise these findings within the broader research landscape, it is essential to note that, while there is indeed a growing and sophisticated body of research on digital health in oncology, much of it, such as recent European initiatives focused on data integration and interoperability (Coppini et al., 2025), addresses clinical data infrastructures rather than communication processes.

However, in paediatric oncology, the literature remains remarkably scarce, particularly regarding digital tools that foster communication among healthcare professionals, primary caregivers, and child or adolescent patients.

Although this review focused on identifying drivers and barriers to digital communication in paediatric oncology, it is essential to acknowledge the potential ethical and safety challenges associated with minors’ use of social media and other open digital platforms.

Recent literature increasingly emphasises the ethical duties of healthcare professionals when using social media and digital platforms in paediatric oncology, highlighting the need for responsible, accurate, and empathetic communication to protect patient trust and well-being (Shukla et al., 2022). In the context of minors, the European MyPal project and others have stressed that digital tools should be designed with strict attention to privacy, participant autonomy, and the emotional safety of children and adolescents (Garani-Papadatos et al., 2022). These ethical standards require systematic safeguards against potential harm, such as exposure to misinformation, infringement of privacy, and unequal access, all of which must be addressed through robust, interdisciplinary strategies. Recent guidelines recommend a transparent, participant-centred approach, integrating diverse perspectives from families, clinicians, and ethicists in the co-design and supervision of digital health applications (Wilke et al., 2025).

Social networks are designed for general audiences and are not appropriate for independent use by children or adolescents, due to privacy concerns, exposure to misinformation, and the lack of expert content validation. Consequently, communication in paediatric oncology through digital means should be mediated and supervised by adults to ensure that the information provided is accurate, age-appropriate, and emotionally safe for young patients. These ethical concerns were beyond the scope of this review but represent a critical area for future research to ensure the responsible design and use of digital communication tools in paediatric oncology.

This gap is clinically significant: hospitals and oncology units have reported, including in the studies reviewed here, an increasing incidence of treatment refusal or non-adherence among young patients, often linked to misinformation or distrust, particularly when families rely primarily on the internet as their primary information source. Yet, no existing digital resource systematically bridges these three communication pillars—clinical professionals (not limited to physicians), caregivers, and patients—within the specific context of paediatric cancer care.

Recent studies have highlighted that non-adherence and treatment refusal among paediatric oncology patients are increasingly linked to online misinformation and a lack of trust—mainly when families rely on digital resources as their primary source of information (Wang et al., 2023; Guidry et al., 2021). Research also demonstrates that knowledge gaps and poorly regulated online information-seeking by caregivers can either prolong diagnostic and treatment delays or, conversely, speed up access to care, depending on the quality of digital resources available (Wang et al., 2023). This duality underscores a pressing need for integrative digital frameworks that systematically connect clinicians, caregivers, and patients—ensuring the delivery of validated, age-appropriate content and supporting shared decision-making (Yamaji et al., 2022).

Therefore, the development of new, integrative digital frameworks in this field represents a necessary and novel contribution rather than a redundancy with existing digital oncology resources.

In line with the non-definitive nature of systematic reviews, the following section outlines the main methodological limitations of the evidence base. It proposes specific avenues for future research to address the gaps identified in this synthesis.

As noted in recent systematic reviews, advancing digital communication in paediatric oncology requires ongoing evaluation of methodological limitations, including variability in technological literacy, fragmented evidence across platforms, and evolving ethical standards (L. Cheng et al., 2021; M. Cheng et al., 2021). Future research should address these gaps by developing inclusive assessment protocols, engaging in participatory co-design with stakeholders, and implementing robust ethical oversight that integrates existing guidelines for digital and AI-based health interventions (Garani-Papadatos et al., 2022).

## 6. Limitations and Future Lines

This study included only articles published between 2004 and January 2023, when the study was carried out; therefore, it is essential to incorporate new developments.

Although we aimed for a comprehensive synthesis, several limitations should be acknowledged.

First, database selection may have introduced selection bias: the search was conducted exclusively in Web of Science, and the additional filter to journals indexed in JCR likely favoured higher-impact outlets while under-representing studies indexed elsewhere (e.g., Scopus, PubMed, PsycINFO) and grey literature (e.g., theses, reports, conference proceedings).

Second, although our eligibility criteria stated no formal language restrictions, reliance on WoS indexing and reviewers’ language competencies likely favoured English-language publications, potentially underrepresenting non-English evidence.

Third, publication bias is possible because we included only peer-reviewed, final-stage articles; studies with null or negative findings and non-indexed outputs are less likely to be captured.

Fourth, our time window (2004–January 2023) may introduce temporal bias: earlier social platforms (e.g., blogs, Facebook) are likely overrepresented relative to newer, fast-growing platforms (e.g., TikTok, Instagram Reels), and platform features evolve rapidly, which can affect the generalizability of our conclusions.

Fifth, the search string and controlled vocabulary—despite being developed via PICOS and PRISMA—may not have captured all relevant synonyms (e.g., platform-specific or colloquial terms), which could have led to omission of eligible studies.

Sixth, the included literature exhibits substantial methodological and contextual heterogeneity (designs, populations, outcomes), which precluded meta-analysis; therefore, our integration is narrative/descriptive and subject to classification subjectivity (drivers vs. barriers). We mitigated this through independent double coding, consensus resolution, and senior supervision, yet residual subjectivity cannot be excluded.

Finally, the risk of bias of individual studies varied. However, the overall strength of evidence for some themes (e.g., platform-specific effects) remains moderate and should be interpreted with caution.

Beyond these methodological considerations, emerging contexts such as the COVID-19 pandemic have reshaped patterns of digital communication in healthcare. Our focus on paediatric oncology led us to exclude other diseases, such as COVID-19; however, future studies could explore the indirect effects of the pandemic on communication dynamics between healthcare professionals, patients, and caregivers. Evidence suggests a notable increase in electronic consultations and health-related interactions on social media platforms such as *X (Twitter)* (M. A. Hansen et al., 2023; Johnson et al., 2020). Understanding these changes may clarify how digital communication can further improve care and support for children and adolescents with cancer (Utengen et al., 2017).

In addition, future research should also examine the ethical and safety challenges associated with digital communication involving minors. Social media and open digital platforms were designed for general audiences and are not appropriate for independent use by children and adolescents. Therefore, new studies should focus on developing moderated and secure communication frameworks that mediate and supervise information, with adults— including caregivers and healthcare professionals—monitoring to ensure accuracy, reliability, and emotional safety for paediatric patients.

Finally, future research should address the development of refined models for filtering scientific information and tools to improve information management and usability (Rothmund et al., 2022), particularly for non-expert patients and caregivers in paediatric oncology. Such advancements will be essential to create trustworthy, user-friendly, and evidence-based digital environments that effectively support the communication needs of paediatric cancer care.

## 7. Conclusions

This review summarises the main evidence synthesised, rather than presenting a final definitive conclusion, of the main drivers and barriers of online scientific communication in paediatric oncology. The findings highlight that digital platforms, such as health websites, mobile applications, and social media, play an essential role in empowering young patients and their caregivers by improving access to information and fostering engagement in care. However, the study also reveals significant challenges, including the persistence of misinformation, lack of credibility, and insufficient adaptation of language and content for children and adolescents.

Understanding these dynamics is key to developing future communication tools that are both educational and emotionally supportive. The integration of healthcare professionals, primary caregivers, and patients within a shared digital ecosystem is a fundamental requirement for trustworthy, age-appropriate communication.

Ultimately, this review contributes to the conceptualization of digital communication in paediatric oncology by emphasising the need for ethical, evidence-based, and supervised digital environments. These environments should strengthen understanding, trust, and adherence among paediatric patients and their families, fostering more effective collaboration with healthcare professionals.

## Figures and Tables

**Table 1:** Inclusion and exclusion criteria (Own elaboration, 2023).

Criteria	Inclusion Criteria	Exclusion Criteria
Target	Children, teenagers with cancer, and their tutors.	Studies whose target population are patients with other kinds of diseases or adults with the same characteristics.
Intervention	A complex understanding of science through platforms addresses these factors: paediatric oncology, digital platforms, ordinary language, and science communication.	Any other intervention that was not a complex understanding of science through digital platforms.
Comparator	Usual communication with the sanitary, usual use of digital platforms, science databases.	Any other type of comparison or intervention.
Outcome	Understanding of scientific knowledge in cancer.	Any other type of knowledge.
Study design	Systematic review, questionnaires, structural equation model with multiple variables, meta-analysis, quantitative and qualitative studies.	Clinical trials.
Publication year	From 2004 (The date on which Facebook emerged as the most used social platform to talk about cancer) till 19 January 2023 (The date the search was performed).	The precedent years.
Language	All languages	None
Setting	All settings.	None.

**Table 2:** Final Codebook (Own elaboration, 2023).

Main Code	Operational Definition	Platform
Trust	Perceived reliability in health information exchange	Health Apps, Social Media and Web Platforms
Quality	Assessment of usefulness and accuracy of information	Health Apps, Social Media and Web Platforms
Credibility	Evaluation of sources and content trustworthiness	Health Apps and Social Media
Interaction	User-to-user and user-content dynamic exchanges	Social Media
Exposure	Reach and sharing of health experiences and outcomes	Social Media
Public perception	Social imagery and community discourse regarding disease	Social Media
Ethics	Ethical dilemmas and guidance in digital health	Web Platforms, Social Media and Health Apps
Access	User-friendliness and accessibility of health resources	Web Platforms, Social Media and Health Apps
Safety	Data protection and privacy safeguards in digital health	Web Platforms, Social Media and Health Apps

**Table 3:** Synthesis Matrix of Included Studies (own elaboration, 2023).

Analytical Dimension	Recurrent Theme	Definition/Description	Representative References
Drivers	Social media as a facilitator of science communication	Use of platforms (e.g., X [Twitter], Facebook, Instagram, TikTok) to share oncology-related content, raise awareness, and build supportive communities.	(Warner et al., 2021; Johnson et al., 2020; M. A. Hansen et al., 2023)
	Educational and training initiatives	Digital programmes or online campaigns designed to improve understanding of cancer science, treatment, or prevention among youth and caregivers.	(Cowfer et al., 2023; Rothmund et al., 2022)
	Collaborative or empathic communication	Online forums or interactive tools that promote dialogue between health professionals, patients, and caregivers.	(E. Lee et al., 2022; Utengen et al., 2017)
Barriers	Misinformation and fake news	Circulation of inaccurate, misleading, or non-evidence-based information about cancer, treatment, or prognosis.	(Warner et al., 2021; M. A. Hansen et al., 2023)
	Lack of trust and credibility	Perceived unreliability of online information sources or health professionals’ digital communication.	(Cowfer et al., 2023; Johnson et al., 2020)
	Excessive technical language	Scientific jargon or complex terminology that limits comprehension among paediatric patients or caregivers.	(Rothmund et al., 2022; E. Lee et al., 2022)
	Insufficient age-appropriate adaptation	Absence of content specifically tailored to children or adolescents (e.g., simplified visuals, gamified tools).	(Utengen et al., 2017; Cowfer et al., 2023)

**Table 4:** Topics and codes related to cancer types, as reflected in medical information exchanged on social networks (Gage-Bouchard et al., 2017).

Theme	Code
Treatment protocols and use of health services	Medical procedures and diagnostic tests. Description and suggestion of treatment options. Use of health services/Navigation of the health system Prognosis. Suggested possible diagnoses for symptoms Information/data on childhood cancer.
Side effects and late effects	Description of the child’s side or late effects and suggestions.
Medication Prescription and non-prescription medicines. Their efficacy and side effects, or medication costs and drug approval. (Rupert et al., 2016).	Description, information, and suggestions for the child’s medicines.
Health care strategies	minimising exposure to germs and infections Medication administration Diet and symptom control at home Adapting home and family routines to medical care
Alternative and complementary therapies The following categories of treatments are observed (Rupert et al., 2016): behavioural therapies (e.g., exercise, diet). Alternative therapies (e.g., acupuncture). Medical therapies (e.g., surgery): Discuss recovery from surgery or chemotherapy and insurance coverage. All therapies have their advantages and disadvantages.	Alternative and/or complementary health services/treatments.
Other	Medical Question Patient Health Update

**Table 5:** The most influential aspects of platforms (own elaboration, 2023).

Feature	Platforms That Have This Characteristic
Educational focus in health (Martiniuk et al., 2021).	Cure4kids, Grapes, COMFORTTM SM Communication Curriculum, SIOP, IAEA, E-Cancer, and NSW EviQ.
Multilingual support (Cure4Kids, 2023).	Cure4kids
Interactive and collaborative features such as “trivia” to enhance understanding of diseases and has garnered positive patient feedback following their engagement (Ouyang et al., 2022).	Grapes or World Pediatric Oncology Organizations (SIOP)
Patient-centred communication improving the patient experience (Wittenberg et al., 2018).	COMFORTTM SM communication programme
Patient and caregiver support (CaringBridge, 2023).	CaringBridge
The global reach offers resources and information on a worldwide scale (Martiniuk et al., 2021).	SIOP, IAEA, E-Cancer, and NSW EviQ
Training (Martiniuk et al., 2021) and resources for healthcare professionals (Doherty et al., 2021).	International Atomic Energy Agency (IAEA) or ECHO
Diversity of content and format (Martiniuk et al., 2021).	E-Cancer and Oncolink
Age-group specific focus (Martiniuk et al., 2021).	Australia Canteen
Community and health support (Martiniuk et al., 2021).	Paediatric Oncology Group of Ontario of Canada (POGO)
Virtual clinics and expert collaboration (Doherty et al., 2021).	ECHO
E-learning programmes (Martiniuk et al., 2021).	Oncolink and NSW EviQ
Emotional support spaces (Grace et al., 2019).	Blogs and microblogs

**Table 6:** The most influential aspects of collaborative mHealth apps (Own creation, 2023).

Feature	Platforms That Have This Characteristic
Social networks for healthcare empowerment that Integrates social networking to share experiences and create community (PatientsLikeMe, 2023).	PatientsLikeMe
Healthcare resources tailored to specific audiences (children) (Gilljam et al., 2020).	Sisom
Caregiver-centred support and communication (Wittenberg et al., 2018).	Caregiver Communication about Cancer
Professional moderation for safe and effective participation (Grace et al., 2019).	PatientsLikeMe, Sisom and Caregiver Communication about Cancer
Integration of evidence-based practices (Wittenberg et al., 2018).	Sisom and Caregiver Communication about Cancer

**Table 7:** The most influential aspects of Social Media Content Communities (Own creation, 2023).

Feature	Platforms That Have This Characteristic		Social Media Type
Social Media Content Communities Platforms (Ponce et al., 2022).	YouTube, TikTok and Pinterest		Social Media Content Communities
User-Generated Content (Warner et al., 2022).			Social Media Content Communities
Visual Orientation or and different content formats (Ponce et al., 2022).			Social Media Content Communities
Informative and Instructional (Ponce et al., 2022).			Social Media Content Communities
Global Accessibility (Ponce et al., 2022).			Social Media Content Communities
Interactivity (Warner et al., 2022).			Social Media Content Communities
Variety of Content (Ponce et al., 2022).			Social Media Content Communities
Focus on Thematic Communities (Warner et al., 2022).			Social Media Content Communities
Versatility in Content Types and Multimodal Communication Platforms (Ponce et al., 2022).		Facebook, X and Instagram	Social Media Platforms
Community Building (Nawaz et al., 2022).			Social Media Platforms
Access to Real-Time Content (Ponce et al., 2022).			Social Media Platforms
Interactive Communication Features (Nawaz et al., 2022).			Social Media Platforms
Interactivity and User Engagement (Nawaz et al., 2022).			Social Media Platforms
Broad Reach and Audience Diversity (Ponce et al., 2022).		Facebook and X	Social Media Platforms
Emphasis on visualisation (Ponce et al., 2022).		Facebook and Instagram	Social Media Platforms
Use of Hashtags (Symplur, 2018).		X and Instagram	Social Media Platforms
Multimodal Messaging features and versatility (De Pouplana, 2020; Telegram, 2023; WhatsApp, 2023).		WhatApp, Telegram and WeChat	Social Media Contact Apps
Focus on Security, synchronisation, and Multi-device access (Telegram, 2023; WhatsApp, 2023).		WhatApp and Telegram	Social Media Contact Apps

## Supplementary Materials

The following supporting information can be downloaded at: https://www.mdpi.com/article/10.3390/ejihpe15120242/s1, Supplementary file S1: Preferred Reporting Items for Systematic reviews and Meta-Analyses ex-tension for Scoping Reviews (PRISMA-ScR) checklist.
